# Applications and Market of Micro-Organism-Based and Plant-Based Inputs in Brazilian Agriculture

**DOI:** 10.3390/plants12223844

**Published:** 2023-11-14

**Authors:** Cláudio Roberto Fonsêca Sousa Soares, Anabel González Hernández, Emanuela Pille da Silva, Julia Emanuela Almeida de Souza, Danyella Fernandes Bonfim, Giovani Leone Zabot, Paulo Ademar Avelar Ferreira, Gustavo Brunetto

**Affiliations:** 1 Departamento de Microbiologia, Centro de Ciências Biológicas, Universidade Federal de Santa Catarina, Imunologia e Parasitologia, Campus Universitário Reitor João David Ferreira Lima, Trindade, Florianópolis 88040-900, SC, Brazil; anabelgonzalezher@yahoo.es (A.G.H.); manupille@gmail.com (E.P.d.S.); 2 Confederação de Agricultura e Pecuária do Brasil, SGAN 601 Módulo K, Asa Norte, Brasília 70830-903, DF, Brazil; ju.emanuela@gmail.com; 3 Agricultural Engineer, SHIN CA 9, Lt 13-15, Ed. Porto do Lago, Lago Norte, Brasília 71503-509, DF, Brazil; dfbonfim11@gmail.com; 4 Coordenação Acadêmica, Universidade Federal de Santa Maria, Campus Cachoeira do Sul, Cachoeira do Sul 96521-000, RS, Brazil; giovani.zabot@ufsm.br (G.L.Z.); paulo.ferreira@ufsm.br (P.A.A.F.); 5 Departamento de Ciência do Solo, Universidade Federal de Santa Maria, Santa Maria 97105-900, RS, Brazil; brunetto.gustavo@gmail.com

**Keywords:** plant growth-promoting micro-organisms (PGPM), seeds inoculation, crop development, biological and biochemical pesticides

## Abstract

The use of plant-based and micro-organism-based biological inputs is a sustainable agricultural practice. It promotes a suitable and better utilization of non-renewable resources in the environment. The benefits of using micro-organisms are associated with direct and indirect mechanisms, mainly related to improvements in the absorption and availability of nutrients, resulting in a consequent impact on plant growth. The main benefits of using biochemical pesticides are the promotion of sustainability and the management of resistance to pests and diseases. Although the use of micro-organisms and botanical metabolites is a promising agricultural alternative, they are still primarily concentrated in grain crops. There is a huge opportunity to expand the plant-based and micro-organism-based biological inputs used in agriculture due to the wide range of mechanisms of action of those products. At a global level, several terminologies have been adopted to characterize biological inputs, but many terms used conflict with Brazilian legislation. This review will clarify the classes of biological inputs existing in Brazil as well as present the application and evolution of the market for microbiological and plant-based inputs.

## 1. Introduction

Several inputs of biological origin have been investigated for their benefits in agriculture. The use of these inputs represents a sustainable production alternative, positively impacting the market [1,2]. It results in a promising perspective on the commercialization and application of these products around the world [3]. The benefits normally associated with biological inputs include increased productivity of crops, better use of natural resources, reduced production costs, and environmental impacts [4]. Bacteria that promote plant growth, such as rhizobia and *Azospirillum* [5,6], and arbuscular mycorrhizal fungi (AMF) [7], are well-established biological inputs in the market and are widely used for this purpose. In recent years, *Bacillus* [8], *Trichoderma* [9], and *Beauveria* [10] have been used in the formulation of biological inputs for biological control. In this article, biological inputs based on micro-organisms are explored, notably bacteria and fungi, which have an intimate relationship with plants, contributing to the growth and nutrition of agricultural species of economic importance. Biological inputs based on plants have the potential to act in the biological control of pests and diseases and are also addressed in this review. Furthermore, aspects of Brazilian legislation regarding the classification of inputs of biological origin used in agriculture and the current market are explored.

## 2. Characterization and Application of Micro-Organism-Based Biological Inputs

Microbial biological inputs are composed of plant growth-promoting micro-organisms (PGPM). PGPM can be capable of reducing the use of chemical fertilizers, improving soil quality, and increasing crop yields through direct and indirect mechanisms [11]. The direct mechanisms are essentially related to improvements in nutrient uptake and availability to plants, while indirect mechanisms function in pest and disease control [12].

In recent decades, several concepts have emerged to categorize microbial biological inputs according to the functional activity associated with the mechanism of action of PGPM. PGPM are classified into biofertilizers (enhance nutrient availability in plants), phyto-stimulants (produce phytohormones), rhizo-remediators (degrade pollutants), and biopesticides (produce antimicrobial compounds) [13]. Therefore, within the biofertilizers category, there are grouped biological inputs that contain micro-organisms mainly involved in biological nitrogen fixation, phosphorus, zinc, sulfur, potassium, and iron solubilization/mobilization. Phyto-stimulants stimulate root growth through phytohormone production, with an emphasis on auxins, cytokinins, and gibberellin producers. Biopesticides include micro-organisms capable of controlling phytopathogens from the soil or seeds through antibiosis, enzyme production, or the induction of systemic resistance.

### 2.1. Direct Mechanisms for Promoting Plant Growth

Nitrogen constitutes approximately 80% of the gases in the atmosphere, being the fourth most abundant element in living organisms. However, this abundance is not utilized by plants, which can only make use of combined forms found in the soil in small quantities. Biological nitrogen fixation (BNF) is undoubtedly the most extensively studied direct mechanism, contributing significantly to the total nitrogen supply required by plants. This process can be carried out by free-living bacteria or in symbiosis with plants, involving the transformation of atmospheric nitrogen into a plant-assimilable form through the action of the nitrogenase enzyme complex. Almost 70% of the nitrogen in the form of NH_4_^+^ is derived from legume-rhizobium symbiosis, which can provide up to 90% of the nitrogen required by leguminous crops [14,15]. Among the bacterial genera that perform free-living fixation, notable ones include *Azospirillum*, *Azotobacter*, *Derxia*, *Beijerinckia*, and *Bacillus*. Within the groups forming well-known symbioses, we have *Nostoc*, *Frankia*, and *Rhizobium*, with the latter being the most widely used in the composition of biological inputs around the world.

Bacteria capable of nitrogen fixation that form nodules in legumes are known as rhizobia. This group can be found in seven families of α and β-Proteobacteria, divided into 15 genera. Among the α-Proteobacteria, notable genera include *Rhizobium* [16], *Mesorhizobium* [17], *Bradyrhizobium* [18], *Sinorhizobium*/*Ensifer* [19], *Azorhizobium* [20], *Devosia* [21], *Phyllobacterium* [22], and *Ochrobactrum* [23]. In the β-Proteobacteria, species from the genera *Burkholderia* [24] and *Cupriavidus* [25] can be found.

Due to the notable importance of BNF, research has been encouraged, and various commercial formulations based on bacteria belonging to these genera have been developed. As a result of this process, for example, in Brazil, there is an extensive list of bacterial strains with nitrogen-fixing capability that have been evaluated and recommended, mainly for legumes due to their symbiotic efficiency [26]. Worldwide, biological inputs based on nitrogen-fixing bacteria constitute more than 70% of commercialized fertilizers. Another important group comprises solubilizers, which represent approximately 15% of these products [27].

Phosphorus is the second-limiting element for plant growth after nitrogen. It is abundant in soils in both organic and inorganic forms, but is typically found in low availability of plants. The dynamics of phosphorus in soils are complex due to the phenomenon of phosphorus fixation, which involves the transformation of labile phosphorus into non-labile forms. This mechanism is explained by the strong affinity that phosphorus has for Ca^3+^, Fe^3+^, and Al^3+^ ions. As a result, in most soils, phosphorus exists in insoluble forms, while plants can only absorb the two soluble forms: monobasic (H_2_PO_4_^−^) and dibasic (HPO_4_^2−^) forms [28].

In agriculture, to address this issue, phosphatic fertilizers are used. However, these fertilizers are often lost because they are quickly converted into their insoluble forms. Phosphate-solubilizing micro-organisms have been studied for their ability to enhance phosphorus availability through solubilization and/or mineralization. Solubilization occurs through the production of low-molecular-weight organic acids, such as gluconic acid and citric acid. Several studies demonstrate that inorganic sources of phosphorus, potassium, and other soil nutrients are solubilized by bacterial species like *Achromobacter*, *Agrobacterium*, *Azotobacter*, *Beijerinckia*, *Bacillus*, *Burkholderia*, *Erwinia*, *Flavobacterium*, *Microbacterium*, *Rhizobium*, *Pseudomonas*, *and Serratia*, as well as fungal species like *Aspergillus*, *Penicillium*, *Fusarium*, *Chaetomium*, and *Cephalosporium* [29,30].

On the other hand, mineralization is associated with the production of phosphatase enzymes, with bacteria involved in the mineralization of organic phosphorus considered the primary source of this enzyme’s activity in soils. Organic phosphorus is present in forms such as inositol phosphate, phospholipids, and nucleic acids, with inositol phosphate being the most abundant and dominant form. Organic phosphorus comprises 30–70% of the total phosphorus in agricultural soils and is as important as the pool of inorganic phosphorus in contributing to the available phosphorus for plants [31].

The availability of phosphorus in the soil can also be strongly influenced by the establishment of symbiosis between roots and arbuscular mycorrhizal fungi (AMF) present in the soil. These micro-organisms expand the root absorption area by producing extraradical mycelium, thus contributing to the uptake of immobile elements in the soil, such as phosphorus. They also enhance water absorption under water-deficit conditions. Many factors can affect the benefits of AMF for agriculturally important species, including plant genotypes [7], fungal species/isolates [32], and soil and climatic conditions [33]. This highlights the need to select fungal isolates for symbiotic efficiency specific to the plant species of interest, as well as to evaluate this efficiency under varying soil fertility levels, particularly in assessing phosphorus availability. This is crucial since phosphorus influences the degree of colonization and the benefits for the host plant [7].

Plant hormones are among the most important regulators of plant growth. They have a direct impact on plant metabolism and interfere with stimulating the plant’s defense response against stress. The production of phytohormones by micro-organisms is well-established in the literature. Recent studies have shown that microbial phytohormones can be used to induce systemic plant tolerance to stressful environmental conditions. It is known that approximately 80% of the bacteria inhabiting the rhizosphere produce indole-3-acetic acid (IAA), the most widely studied type of auxin. *Azospirillum* spp., *Azotobacter* spp., *Aeromonas* spp., *Burkholderia* spp., *Enterobacter* spp., *Pseudomonas* spp., and *Rhizobium* spp. are the main bacterial genera in the rhizosphere capable of synthesizing this phytohormone [34,35,36,37]. IAA acts on the division and elongation of plant cells, particularly by stimulating the emergence of lateral roots and root hairs. This aspect is of high importance for nutrient absorption and water acquisition, thereby mitigating the effects of abiotic stresses. Furthermore, the production of this phytohormone by micro-organisms plays an important ecological role, as it helps establish a communication signal with plants, facilitating the mutualistic benefits for micro-organisms through the association. Micro-organisms capable of producing IAA are already present in commercial inoculants, contributing to increased productivity of economically important grasses in Brazil [1].

Cytokinins are also an important group of plant hormones that play a crucial role in maintaining cell proliferation and differentiation and are also associated with the inhibition of premature leaf senescence. Zeatin is the most studied cytokinin. The production of these substances occurs at the root tips and is subsequently transported through the xylem. Cytokinins produced by plant growth-promoting bacteria at low hormonal levels have a stimulating effect on plants. This phytohormone can also participate in root nodule organogenesis, promoting their development. It was observed that cytokinin production in bacterial species of the genera *Arthrobacter*, *Bacillus*, *Azospirillum*, and *Pseudomonas*, stimulated the root development of associated plants [38].

Gibberellins are another significant group of plant growth regulators. They are known as regulators of reproductive organs and fruit formation. There are more than 89 types of gibberellins, with gibberellic acid being the most common and well-studied. They play a crucial role in cell division and elongation, seed dormancy, and germination, as well as in controlling the photosynthesis rate and chlorophyll content in plants. *Acinetobacter*, *Alcaligenes Azospirillum*, *Azotobacter*, *Bacillus*, *Bradyrhizobium*, *Burkholderia*, *Pseudomonas*, *Pantoea*, and *Rhizobium* are some of the bacterial genera where the production of this phytohormone has been observed [39,40]. This production has also been noted in endophytic fungi, such as *Aspergillus fumigatus* [41]. Several studies have shown that gibberellic acid stimulates plant growth and development under various abiotic stress conditions [42].

Ethylene is a hydrocarbon gas widely used in agriculture. It acts to inhibit growth, seed germination, the onset of flowering, and fruit ripening. Under stress conditions, plants produce ethylene in elevated concentrations, which can lead to plant senescence [43]. Although ethylene is essential for the growth and development of plant species, it is necessary to regulate its harmful levels [44]. Among other adverse effects of ethylene, it has been noted that it can act as an inhibitor of nodulation in legumes. Some PGPM possess mechanisms enabling control through the production of enzymes like rhizobitoxin and 1-aminocyclopropane-1-carboxylate (ACC) deaminase, which participate in the biosynthetic pathway of ethylene [45].

Rizobitoxin is an enol-ether amino acid produced by bacteria of the genus *Bradyrhizobium*. For a long time, it was considered a phytotoxin as it could cause chlorosis in soybean leaves. Later, it was discovered that this substance also strongly inhibits ACC synthase, an enzyme that is a key factor in the biosynthesis of ethylene. Rizobitoxin plays a positive role in the establishment of symbiosis between *Bradyrhizobium elkanii* and symbiotic legumes, acting to reduce the endogenous levels of ethylene in the roots. Currently, it is known that rhizobitoxine production is restricted *to B. elkanii* strains [46,47]. For this reason, several studies have questioned what mechanisms other microbial species might use to counteract the deleterious effects of ethylene on plants. As a result, the enzyme ACC deaminase (E.C. 3.5.99.7) was discovered, responsible for the irreversible conversion of ACC, the immediate precursor of ethylene, into ammonia and α-ketobutyrate [48]. This enzyme has been detected in various microbial genera, but its distribution is not uniform as it can be absent in organisms of the same genus and species. According to Glick [49], there are at least two direct consequences resulting from the decrease in ACC levels within the plant: a reduction in ethylene levels and the subsequent decrease in growth inhibition and cell proliferation. Consequently, plants that establish symbiotic relationships with growth-promoting bacteria possessing ACC deaminase enzyme activity may develop longer roots and potentially exhibit higher aboveground growth.

### 2.2. Indirect Mechanisms for Promoting Plant Growth

Some micro-organisms can also promote plant growth indirectly by preventing or reducing damage caused by phytopathogens. Among the most studied indirect mechanisms are competition for nutrients and space, the synthesis of siderophores, antibiotics, hydrogen cyanide (HCN), toxins, bacteriocins, and hydrolytic enzymes. Additionally, the synthesis of volatile organic compounds and plant hormones, such as salicylic acid and jasmonic acid, and the modulation of ethylene levels contribute to systemic resistance in many plant species.

Competition for nutrients and space is an important biocontrol mechanism. This occurs because both beneficial micro-organisms and phytopathogens can colonize the same niches and utilize the same nutrients [50]. Biological control through nutrient competition occurs by locally and temporally increasing highly competitive strains during critical stages of the pathogen’s life cycle. The control takes place without any direct interaction between the two organisms, as the antagonist acts by producing enzymes that more rapidly degrade complex organic matter, utilizing simple carbohydrates and amino acids more quickly, or producing siderophores in the case of iron competition [51]. Thus, these strains modulate the growth environment in the target niche, making the conditions less favorable for the pathogen’s development.

Spadaro and Droby [50] described how the use of fast-colonizing yeasts is an effective mechanism against pathogen invasion. In their study, they analyzed the competition processes between *Pichia guilliermondii* and the pathogens *Penicillium digitatum*, *P. expansum*, *Botritis cinerea*, or *Colletotrichum* spp. on wounds of different fruits. Yeasts, as unicellular organisms, are capable of rapidly multiplying under favorable conditions in nutrient-rich fruit wounds, making it challenging for the pathogen to establish itself.

Siderophores are chelating secondary metabolites that function by binding to Fe^3+^ and transporting it across the bacterial cell membrane under conditions of limited iron availability [52]. Siderophores produced by plant growth-promoting bacteria act as biocontrol agents, as their high affinity for Fe can prevent phytopathogens from acquiring the necessary amount of this element, thus limiting the pathogen’s infection capacity [53,54]. Various species within the bacterial genus *Pseudomonas* have frequently been described as siderophore producers, reducing the population of pathogens in the rhizosphere [55,56]. Another example was described by Segarra et al. [57], who found that the fungus *Trichoderma asperellum* can produce siderophores, thereby controlling diseases caused by *Fusarium* sp.

Antibiosis is the ability to produce antimicrobial compounds that suppress or reduce the growth and/or proliferation of phytopathogens. These are mostly products of secondary metabolism belonging to heterogeneous groups of low molecular weight organic compounds, including antibiotics, volatile compounds, and cell wall-degrading enzymes, among others [51,58]. Characterization studies and elucidation of the mode of action of these compounds have formed the basis for the selection and commercialization of some strains for biological control. The production of these metabolites has been described in bacterial genera such as *Agrobacterium*, *Bacillus*, *Pantoea*, *Pseudomonas*, *Serratia*, *Stenotrophomonas*, and *Streptomyces*, and in fungal genera such as *Trichoderma*, *Purpureocillium*, *Boletus*, *Suillus*, *Chroogomphus*, *Xerocomus*, *Pisolithus*, *Russula*, and *Scleroderma* [59]. Some of the most studied examples include iturin, surfactin, fengycin, 2,4-diacetylphloroglucinol (DAPG), pyrrolnitrin, and phenazine from bacteria [60,61], and trichodermin, trichodermol, gliovirin, gliotoxin, viridin, and leucinostatins from fungi [62,63]. Among the prominent lytic enzymes are chitinases, cellulases, xylanases, pectinases, glucanases, lipases, amylases, arabinases, and proteases [58], which can lyse the cell wall of phytopathogens such as *Botrytis*, *Fusarium*, *Phytophthora*, *Pythium*, *Plectosporium*, *Rhizoctonia*, *Sclerotium*, and *Verticillium*, among others [64,65].

PGPM can also trigger a phenomenon in plants known as induced systemic resistance (ISR), similar to systemic acquired resistance (SAR), where plants activate their defense mechanisms, playing a significant role in suppressing pathogens. This defense mechanism does not target specific pathogens, making it particularly interesting for controlling diseases caused by various agents. Bacterial molecules like the O-antigenic side chain of bacterial LPS, flagellar proteins, pyoverdine, chitin, β-glucans, cyclic lipopeptide surfactants, and salicylic acid have been described as signals for inducing this type of systemic resistance [66]. It is relevant to highlight that categorizing PGPM into groups based on their mechanisms of action is a methodological exercise to meet regulatory requirements for registration in Brazil. In reality, in nature, interactions occur dynamically, and many times a micro-organism possesses different mechanisms of action that work simultaneously, resulting in biocontrol.

PGPMs can also indirectly benefit plant growth through their action in enhancing tolerance of various abiotic stresses. Among these, drought tolerance [67], salinity [68], and soil contamination with organic chemicals (pesticides and petroleum-derived hydrocarbons) [69] and inorganic chemicals (metallic elements and metalloids) [70] stand out with a favorable role in the bioremediation processes of contaminated soils [71].

## 3. Definition and Regulation of Biological Inputs in Brazil

In Brazil, biological inputs fall under specific legislation according to their mode of action, and the definition of registration criteria considers their agronomic efficiency as well as potential environmental and human health risks. The fertilizer law number 6.894/1980 [72] and decree number 4.954/2004 [73] include products that act in plant nutrition and make it clear that the products must be free from agrochemical substances. Among these products are fertilizers, defined as mineral or organic substances, natural or synthetic, that provide one or more plant nutrients. Regarding biological inputs considered as fertilizers, Brazilian legislation adopts two terminologies defined as follows:

(i) *Inoculants*: products containing micro-organisms that have a favorable impact on plant growth [26].
(ii) *Biofertilizers*: products containing an active ingredient or organic agent, free from agrochemical substances, capable of acting directly or indirectly on all or part of cultivated plants, enhancing their productivity, regardless of their hormonal or stimulant value [74].

Unlike many countries, in Brazil, biological inputs called “inoculants” are essentially composed of micro-organisms that directly promote plant growth. In this context, biofertilizers will not be addressed in this manuscript due to the definition of Brazilian legislation. It is important to note that these products are regulated and supervised solely by the agricultural authority, the Ministry of Agriculture and Livestock (MAPA/Brazil).

Biological inputs that have a mode of action in pest and disease control (also known as biodefensives) are regulated under the pesticide law number 7.802/1989 in Brazil [75]. This law encompasses biological control products, semiochemicals, biochemicals (hormones and growth regulators), microbiological agents, biostimulants, and biological control agents (invertebrates) [75]. According to Brazilian legislation, pesticides and related substances are defined as products and agents of physical, chemical, or biological processes intended for use in agricultural production, storage, and processing of agricultural products, pastures, the protection of native or planted forests, and other ecosystems, as well as urban, water, and industrial environments. Their purpose is to alter the composition of flora or fauna to protect them from the harmful action of living organisms considered pests. Additionally, substances and products used as defoliants, desiccants, growth stimulants, and growth inhibitors are also classified as pesticides.

Thus, according to Brazilian legislation, chemical, physical, and biological active agents are included in the definition of pesticides. Pesticides originating from chemical processes are composed of synthetic chemical substances, while those originating from physical processes can control pests through methods such as suffocation, crushing, burning, drainage, flooding, and temperature. Biological control agents are part of a strategy that involves releasing and applying natural enemies to prevent pests from reaching levels of economic damage [76]. Natural enemies are categorized into two groups: biological control agents, including predators and parasitoids; and microbiological agents, which are fungi, viruses, and bacteria. These microbiological agents also play a significant role in controlling soil and foliar diseases.

Products classified as pesticides are separated into different categories based on the type of substance used, as presented in Table 1. The active substances are mostly of low toxicity and act on the elimination of the target pest without harming the environment, allowing beneficial insects (natural enemies) to remain in the crop. Formulations derived from these organisms and substances are developed and validated as biostimulants or for combating diseases and pests found in the field.

The Brazilian legislation on pesticides deals with highly regulated items as established in law number 7.802/1989 [75], which provides safety to the evaluation and registration process of these products in the country. In addition to the involvement of MAPA as the registering authority, the law includes the health authority (National Health Surveillance Agency—ANVISA) and the environmental authority (Brazilian Institute of the Environment and Renewable Natural Resources—IBAMA). In this context, MAPA evaluates the product’s effectiveness in the field, considering its practicality and agronomic efficiency. ANVISA is responsible for evaluating the toxicological dossier, assessing the product’s toxicity to the population, and determining under which conditions its use is safe. Meanwhile, IBAMA assumes the responsibility of evaluating the environmental dossier, which characterizes the product’s potential to cause environmental impacts.

Therefore, there is a complementary relationship observed among microbial-based biological inputs for use in Brazilian agriculture. These products represent a growing market, complementing or even replacing the use of chemical inputs, including both pesticides and fertilizers. However, it is important to highlight that the adoption of biological products is closely linked to the adoption of integrated pest management (IPM). Furthermore, it is important to emphasize that both crop rotation and intercropping play a pivotal role in promoting the diversity of plant species in the agricultural environment. This results in the enrichment of soil microbial activity, which contributes to the reduction in pest pressure while simultaneously enhancing soil quality [77].

**Figure 1:**
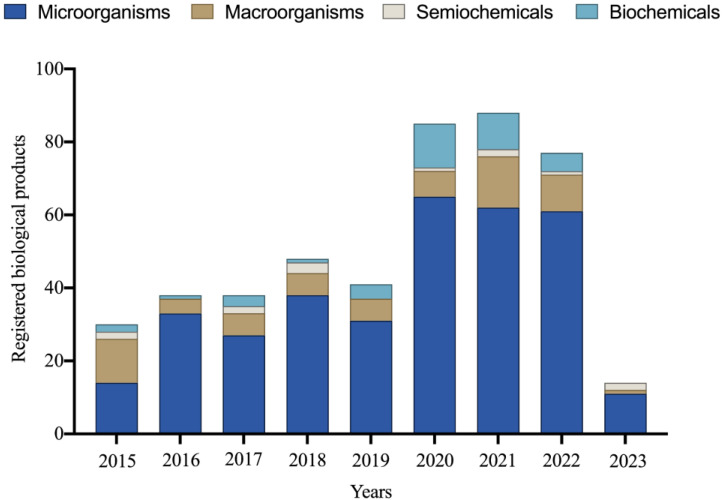
Number of biological products registered between 2015 and 2023 for various Brazilian crops. Data obtained in 2023.

Between 1991 and 2023, 616 new products based on micro-organisms, macro-organisms, semiochemicals, and biochemicals were officially registered for various Brazilian crops [78]. The importance of micro-organism-based products is remarkable, constituting 65% of the registered products during this period. It is worth noting that in the last nine years, there has been significant growth in the Brazilian market for products utilizing micro-organisms, representing an impressive 342% increase in new registrations in the biological products category (Figure 1).

## 4. Biological and Biochemical Pesticides

Biopesticides include micro-organisms (biological pesticides) and plant extracts and essential oils (biochemical pesticides) as active ingredients to suppress one or more plant pathogens. The technology readiness level has increased over the years in this area due to increased investment in science and the transfer of technology to productive industries. Therefore, species of micro-organisms that are stable for formulations have been discovered and used in Brazil, promoting sustainable agriculture with enhanced ecological benefits. The combination of biological and biochemical defenses can enhance the effectiveness of pest control, providing a more efficient and powerful outcome.

### 4.1. Biological Pesticides

Bt (*Bacillus thuringiensis*) is the most well-known microbial pesticide, which has been on the market for over 70 years and accounts for more than 50% of the market share [79]. It is estimated that the use of biopesticides around the world is increasing by almost 10% each year [80]. The successful use of Bt and other microbial species has led to the discovery of many microbial species and strains. *Beauveria bassiana* and *Metarhizium anisopliae* are the most well-known fungi for controlling insect pests [10]. These fungi possess high virulence, environmental stress resistance, and prolific sporulation capacity. Overall, fungi are the primary insect pathogens, causing approximately 80% of insect diseases. They can penetrate the insects’ integument, affecting them at all stages of development [81].

Bionematicides include the use of organisms for nematode control, such as the bacteria *Pasteuria penetrans*, the fungus *Paecilomyces lilacinus*, and even entomopathogenic nematodes from the genera *Heterorhabditis* and *Steinernema*. They act as obligate parasites, generally entering their hosts actively through natural openings, such as spiracles, mouths, and anus [82]. The organisms release their symbiotic bacteria that produce substances induced by apoptosis or necrosis in host cells, triggering their death [83]. The root-knot nematodes *Meloidogyne javanica*, *Meloidogyne incognita*, *Meloidogyne arenaria*, and *Meloidogyne hapla* are the most devastating pests of crops [84].

According to MAPA, two biological acaricides are registered in Brazil. The first one presents the commercial name Barkmax and has the mite *Neoseiulus barkeri* as the active ingredient. The main target to control is the broad mite, *Polyphagotarsonemus latus*. The second one presents the commercial name Bovenat and has the fungus *Beauveria bassiana* as the active ingredient. The main target to control is the mite, *Tetranychus urticae*. For insecticides, fifteen biological products are registered, of which the main active ingredients include *Metarhizium anisopliae*, *Beauveria bassiana*, *Cryptolaemus montrouzieri*, *Bacillus thuringiensis*, *Trichogramma pretiosum*, *Chrysoperla externa*, and *Trichogramma galloi* [85].

Fungicides have been developed as well. Fusarium wilt in cucumber (*Cucumis sativus* L.) was controlled by bio-organic materials (cellulolytic enzymes) produced by *Aspergillus niger* and paper waste as substrates [86]. Twelve endophytic *Trichoderma* spp. from healthy rice leaves were tested for their antagonistic activity against rice disease. Four *Trichoderma asperellum* strains and two strains each of *Trichoderma harzianum*, *Trichoderma koningiopsis*, *Trichoderma longibrachiatum*, and *Trichoderma virens* were identified, and their biocontrol activity was effective against dirty panicle disease in field trials, reducing the disease by 60% when applied at 10^6^ spores mL^−1^ three times at the panicle initiation, flowering, and milk stages [9].

Regarding herbicidal effects, fungal broth obtained by submerged fermentation using the fungus *Diaporthe schini* showed an average inhibition of 40% of weed growth of *Amaranthus viridis*, *Bidens pilosa*, *Echinocloa crusgalli*, and *Lollium multiflorum* [87], which are weeds that attack many crops in Brazil, especially soybean, corn, and cotton. The fungus *Phoma dimorpha* has also been used in submerged fermentation for the production of broth and concentration by membranes (ultrafiltration, microfiltration, and nanofiltration). The membrane concentration was indicated to be optimistic because phytotoxicity was total (100%) against *Senna obtusifolia* for the retained fraction. An expressive reduction in plant height was achieved for *Echinocloa* sp. and *Amaranthus cruentus* as well [88].

### 4.2. Biochemical Pesticides

**Figure 2:**
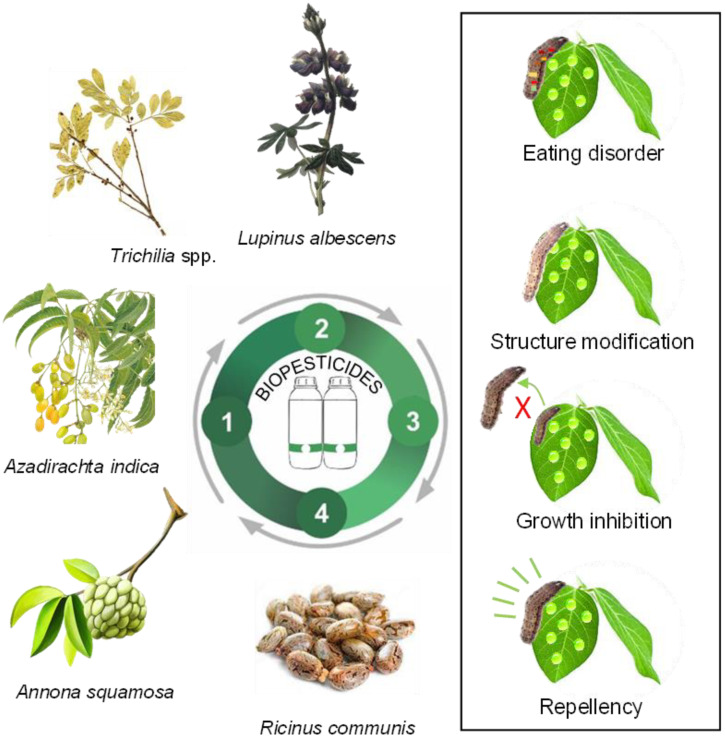
Examples of the mode of action of biochemical pesticides from plants in pest manage-ment for sustainable agricultural production; 1: Prospection; 2: Development; 3: Production; 4: Use and application.

Biochemical pesticides are chemical compounds, such as plant extracts and essential oils, used for pest control because they contain many bioactive substances. It is estimated that only 2400 species have been identified, and only 10% of the compounds found in plant biochemical defenses have been explored, indicating a vast field of scientific research yet to be investigated [89]. Plant-based insecticidal properties are referred to as secondary metabolites, such as phenols, alkaloids, and terpenoids, which are extracted from specific parts of plants such as leaves, flowers, seeds, or roots [90,91]. For instance, biochemical insecticides can induce some modes of action in target pest species, such as feeding disruption, growth inhibition, repellency, and modifications to their structure and physiology (Figure 2).

*Trichilia* spp. (Meliaceae) has attracted interest due to its bioactive phytochemicals that exhibit insecticidal activity. This genus is found in some Brazilian biomes, such as in “Mata Atlantica” and “Pampa”. Extracts obtained from *Trichilia* spp. contain compounds such as limonoids, monoterpenes, steroids, flavonoids, and triterpenes, among others, which affect insect feeding, physiology, and reproductive potential reduction [92]. The extracts demonstrate good efficiency in reducing the larvae of *Spodoptera frugiperda* [93].

Some botanical compounds are commercially available and have shown success against specific groups of agricultural pests. Examples of products are based on neem’s azadirachtin (*Azadirachta indica*), pyrethrin (*Chrysanthemum cinerariaefolium*), and garlic (*Allium sativum*), among others. Table 2 demonstrates plant extracts and oils that have been explored in scientific research as promising plant species containing effective biotics against pests. These plant extracts and oils contribute significantly to IPM programs for sustainable agriculture.

Plant secondary metabolites are being studied for the production of fungicides [100]. Bioactive compounds derived from plants are considered more selective and abundant. For the management of phytopathogenic fungi, some botanical fungicides are marketed, having active ingredients such as pyrethrins, curcuminoids, azadirachtin, and cinnamaldehyde [105,106].

Biochemical pesticides also include substances such as eucalyptol, nicotine, citronella oil, piperine, and terpenoids. These active ingredients are extracted from several botanical species and can act through inhibition of protein synthesis, hyperexcitability, inhibition of nucleic acids, disruption of the nervous system, synapses of axons, production of neurotoxins, modification of sodium channels, and loss of coordination [69,107,108].

## 5. Biological Input Market

The increasing demand for food produced with fewer agrochemicals (or their absence) and the emphasis on using agricultural products that are less harmful to the environment are global trends that are also reflected in Brazil. In this context, the biological input market is rapidly growing worldwide. Micro-organisms, insects, biochemicals, and semiochemicals are already recognized as significant allies for farmers in crop protection. Simultaneously, the reduction or even substitution of chemical inputs with biological inputs offers more than just saving currency; it represents a significant reduction in the consumption of fertilizers and chemical pesticides that rely on fossil energy for their production.

**Figure 3:**
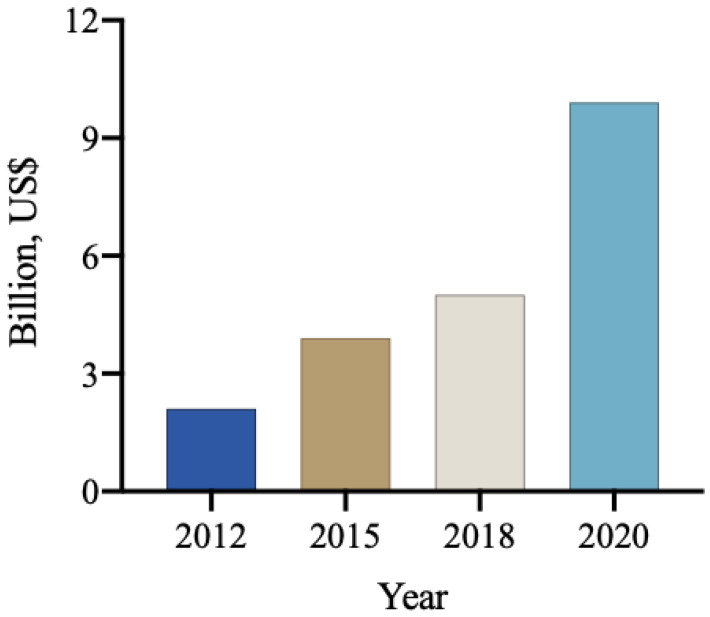
The annual revenue (billions of USD) from the commercialization of biological inputs worldwide [111].

The global market for biological products in agriculture, encompassing biodefensives, inoculants, biostimulants, and biofertilizers, was estimated to be approximately 9.9 billion USD in 2020. Among these, biological products for pest and disease control accounted for 5.2 billion USD [109], with a projection to reach 8 billion USD by 2025. Notably, the use of these products for pest and disease control has been experiencing an annual growth rate of approximately 14% (Figure 3). It is anticipated that the global biological input market will maintain a high growth rate, with projections exceeding 10 billion USD for biodefensives and 3 billion USD for biostimulants [110].

Currently, Europe and the United States lead the market for biological products, each with over 30% of the market share. Latin America ranks third, with Brazil being a prominent player, where the adoption of biological products for pest control is growing rapidly. In turn, Latin America has shown the best performance in terms of growth rate, with a compound annual growth rate (CAGR) of 18% from 2015 to 2020. The region is expected to continue experiencing high annual growth rates, particularly driven by the growth of the biological control market in Brazil.

It is important to mention that Brazil holds a competitive advantage compared to other countries because it extensively employs biological control in large areas, particularly in grain production, which has been growing significantly. This trend stems from the need for producers to enhance resistance management and soil quality. Additionally, several factors contribute to this scenario. Brazil’s agriculture is situated in the only region globally with the potential for expansion in terms of area due to limitations in other parts of the world, armed conflicts, or insoluble constraints related to water and nutrient supply. Another avenue for growth lies in intensifying cultivation, both spatially and temporally, through shorter crop cycles. This implies that over the next 30 years, Brazil will consolidate its role in agricultural production, becoming even more responsible for supplying the increasing global population with food.

In this scenario, Brazil has gained significant importance in production and has become a major consumer of inputs such as seeds, energy, fertilizers, and chemical pesticides. In Brazil, 76% of the ingredients used for manufacturing these products are imported from supplying countries like China, Russia, Canada, and India, which currently face challenges in maintaining their supply pace. This situation leads to a reduction in the supply of fertilizers and agricultural chemicals, resulting in a price increase of over 200% in the 2021/2022 crop season.

Despite the challenges posed by this dependency on inputs and its growth due to intensified cultivation, they present a significant opportunity for the development of solutions, such as domestic biological inputs, fostering a virtuous cycle of product and service development, company creation, and wealth generation for Brazil. According to data from CropLife Brazil [78], the biological input sector is already being used on over 50 million hectares in agricultural production in 2022 and continues to grow significantly. In the case of horticultural crops, the use of biological inputs reaches high rates, but the annual growth in these areas is not as pronounced as observed in crops like soybean, sugarcane, corn, cotton, and coffee.

### 5.1. Inoculants

**Figure 4:**
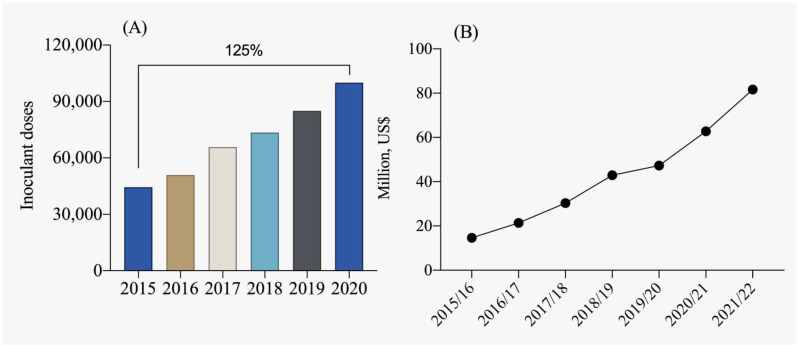
Evolution of the inoculant market in terms of doses used (**A**) and commercialization (**B**) in millions of USD per crop season [113].

In the context of Brazilian legislation, the term “inoculant” is defined as a substance containing micro-organisms that enhance plant development by promoting biological nitrogen fixation, mineralization, and phosphorus solubilization, as well as potassium solubilization, and overall plant growth. Internationally, these products are also classified as biofertilizers. Currently, Brazil is one of the leading producers of inoculants, with production exceeding 75 million doses (Figure 4A). Inoculants for biological nitrogen fixation dominate this segment, representing approximately 80% of the market, followed by phosphorus solubilizers at approximately 15% and other growth-promoting inoculants at approximately 5% [112]. From the 2015/2016 crop season to the 2021/2022 crop season, there has been an almost fourfold growth in the market, highlighting the importance of using plant growth-promoting micro-organisms in Brazilian agriculture (Figure 4B). According to ANPII [113], approximately 97% of total inoculants used in Brazil are produced domestically, while the remaining 3% are imported, mainly from Argentina and Uruguay.

**Figure 5:**
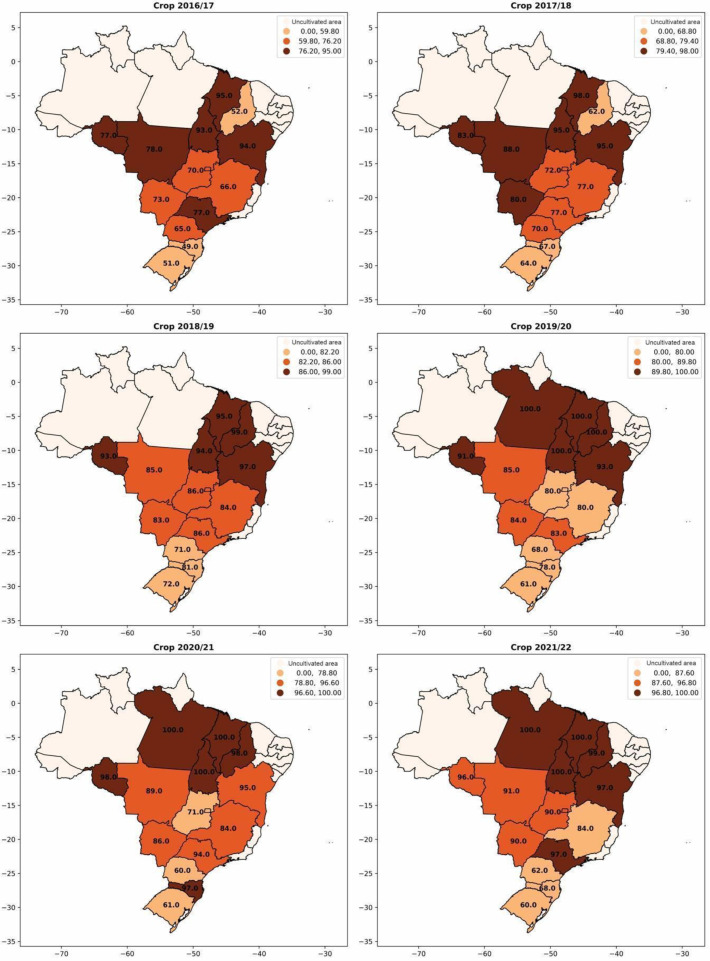
Percentage of adoption of *Bradyrhizobium*-based inoculants for biological nitrogen fixation per area in soybean cultivation from the 2016/2017 season to the 2021/2022 season.

The use of inoculants for biological nitrogen fixation with bacteria of the genus *Bradyrhizobium* has also experienced significant growth in recent crop seasons (Figure 4). In the 2016/2017 crop season, approximately 68% of the soybean cultivated area used inoculants containing *Bradyrhizobium*. However, in the 2021/2022 crop season, this number increased to 88%, representing a significant growth of 18%. In Figure 5, the evolution of inoculant use for biological nitrogen fixation by each Brazilian state is observed. The expansion of soybean cultivation to the northern and northeastern regions of Brazil and its adoption in almost the entire area confirms the relevance of inoculation for increasing soybean productivity and reducing dependence on imported nitrogen fertilizers.

**Figure 6:**
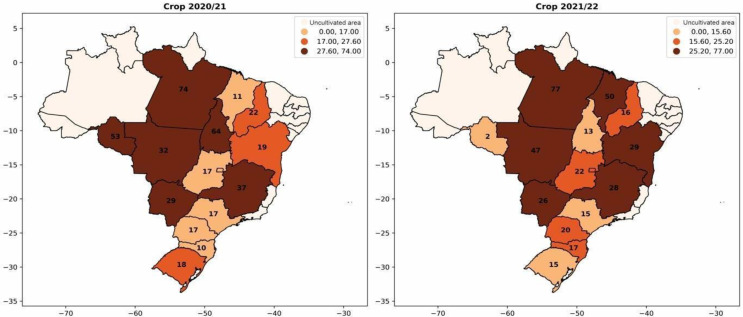
Percentage of adoption of *Bradyrhizobium* and *Azospirillum* coinoculants per area for soybean cultivation from the 2020/2021 season to the 2021/2022 season.

Soybean coinoculation is a technology that has been spreading in Brazil. This primarily involves the simultaneous application of *Bradyrhizobium* and *Azospirillum*, and the results have been increasingly positive for farmers. In 2013, the first commercial product for soybean co-inoculation was registered [1,5]. Recently, in 2021, a comprehensive meta-analysis was conducted based on 51 scientific publications and data from 39 field trials. This analysis statistically confirmed the benefits of coinoculation, revealing average increases of 11% in root mass, 5.4% in nodule number, 10.6% in nodule mass, 3.6% in grain yield, and 3.2% in grain nitrogen content compared to exclusive inoculation with *Bradyrhizobium* spp. [6]. In Figure 6, a significant increase in the use of coinoculation in soybean cultivation is observed. This is reflected in the expanding adoption of simultaneous inoculation of the two bacteria each season, reaching nearly 30% adoption. In Mato Grosso (Brazil), there was a 15% increase in coinoculation usage in 2021 compared to 2020.

Modern agriculture has been advocating for the adoption of environmentally friendly, low-input (sustainable) environmental protection programs, leading to the search for new biological inputs with mechanisms of action beyond those already established, such as FBN. In this context, arbuscular mycorrhizal fungi, which were neglected for many years, have been demonstrating significant potential for application. In general, the benefits of mycorrhizal association for agricultural production are most pronounced under conditions of adversity for plant growth, such as cultivation in low-fertility soils, the presence of toxic elements like aluminum and heavy metals, and occurrences of water deficits [7,31].

Unlike traditional biofertilizer formulations based on nitrogen-fixing bacteria, mycorrhizal inoculants typically consist of presterilized substrates containing selected AMF propagules (spores and hyphae) and mycorrhizal roots. Despite the significant technological potential of AMF for agricultural systems, there are still obstacles to their widespread application due to the limited availability of commercial inoculants and the need for a large volume of inoculants for extensive cultivation areas. In Brazil, the market for AMF-based inoculants is quite nascent, as the registration of the first commercial product in the country dates back to 2018. Formulations based on AMF species like *Glomus mosseae*, *G. aggregatum*, *G. etunicatum*, *Rhizophagus intraradices*, and *R. irregularis* are available in the Brazilian market for use in sugarcane, corn, and soybean production. However, there is still no official data regarding the volume of these fungal inoculants sold in the country.

### 5.2. Biodefensives

**Figure 7:**
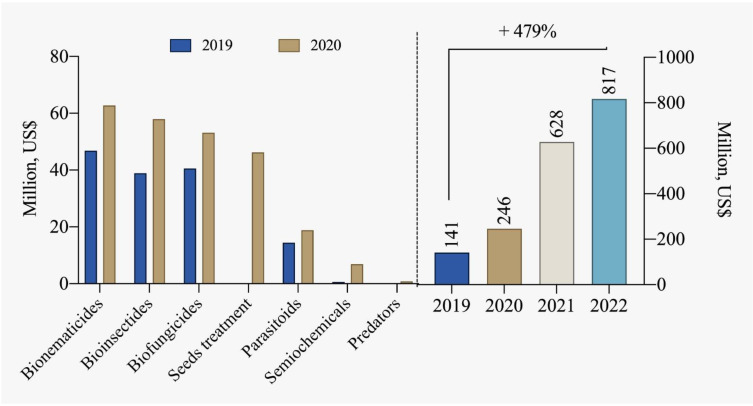
Comparison of segmented commercialization of biological inputs and the total marketed between 2019 and 2022 [78,114].

The number of registrations for biological inputs for pest and disease control in Brazil was 107 products in 2013. Currently, this number has significantly increased to 526 products [85]. This growing trend in the sector is promising and should be supported and encouraged, especially considering market projections indicating a potential of up to 4.2 billion USD for biological controllers by 2030. According to data from CropLife Brazil [78] and ABCBio [114], the biological products industry recorded sales of over 817 million USD in 2022 in Brazil, marking a substantial increase of 479% compared to 2019 (Figure 7).

Bionematicides, products designed for nematode control, are the best-selling active ingredients in the country (Figure 6), primarily for soybean and sugarcane crops. In the central-northern region of the country, there are areas where nematode occurrence levels already exceed 60% of the cultivated area in these fields. Other notable segments include bioinsecticides and biofungicides primarily used in seed treatment. Furthermore, the demand for bioinsecticides has also been growing significantly, aiming to control piercing-sucking insects. The industry has already introduced innovative technologies to address these issues with products based on entomopathogenic fungi, parasitoids, bacteria, among others. The development of biological products applied through seed treatment is also highly beneficial for Brazilian farmers. In addition to being effective, these products offer significant cost benefits and promote increased microbiological activity in the soil, leading to productivity gains.

**Figure 8:**
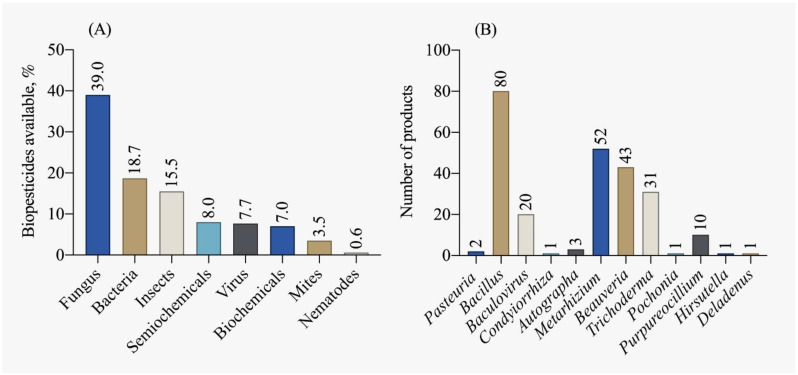
Biopesticide products available in Brazil by (**A**) main classes and (**B**) active ingredient [78].

According to the IBAMA [115] report on the commercialization and production of biopesticides, there was a 47.2% increase in production, a 20.4% increase in imports, and a 50.4% increase in Brazilian sales in 2019 compared to 2018. The majority of biopesticides traded are of microbiological origin (fungi and bacteria), as shown in Figure 8A. The five most registered biological control agents, accounting for 50% of the registrations in the country, are: *Beauveria bassiana*, *Bacillus amyloliquefaciens*, *Metarhizium anisopliae*, *Bacillus subtilis*, *Beauveria bassiana* and *Metarhizium anisopliae*. Currently, most available biocontrol products are dominated by micro-organisms of the *Bacillus* genus, followed by *Metarhizium*, *Beauveria*, and *Trichoderma*, as depicted in Figure 8B.

The investment by the biopesticide industry in the development of new technologies is keeping pace with market growth. In the last 3 years, there has been a 45% growth in the total number of authorized biopesticides for use in Brazil. The significant advancement in the number of registered biopesticides is noteworthy. As of March 2022, there were 502 products with active registration, covering a wide range of categories such as bioinsecticides, biofungicides, bionematicides, pheromones, allelochemicals, growth regulators, and bioacaricides targeting approximately 200 biological targets [85].

**Figure 9:**
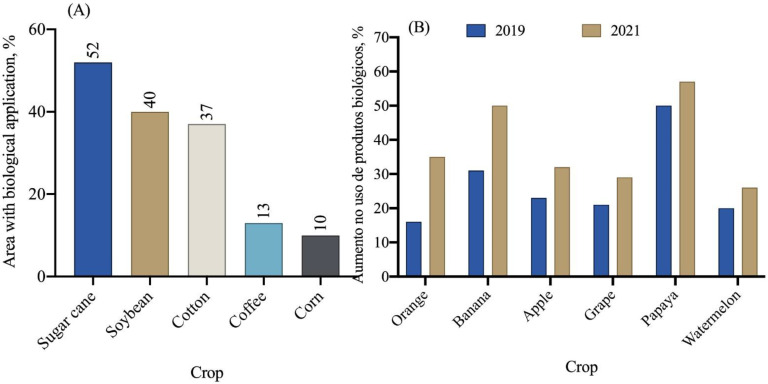
Percentage of area with the use of biopesticides (**A**) and increase in the use of biological products in fruit crop areas (**B**) [78].

In general, the area cultivated in Brazil with various crops reached 74.5 million hectares in the 2021/2022 crop year, according to the National Supply Company—CONAB [116]. Among the crops cultivated in the country, sugarcane utilizes biopesticides for pest and disease control in approximately 52% of the cultivated area (Figure 9A). Additionally, the soybean and cotton crops also show significant use of biopesticides in their cultivation areas.

In recent years, a significant increase in the use of biological products has been observed in citrus, banana, apple, grapevine, papaya, and watermelon crops (Figure 9B). In 2019, the average use of biological products in these crops was 25%, while in 2021, this number increased to 39%, representing a 14% increase in just one year. Among the fruit crops, oranges, bananas, and papaya are the ones that use biological products the most (Figure 9B).

The extraordinary growth of the biopesticide market for pest control in Brazil is associated with several political, commercial, and technical reasons [109]. In the country, significant adoption of biopesticides has been highlighted in crops such as soybean, corn, sugarcane, cotton, and coffee, with an average adoption rate of biological control of approximately 18%. This reflects an increase and diversification in the supply of new products/organisms. Additionally, the intensification of IPM practices in Brazil, combined with higher adoption of agricultural technologies and precision farming tools, has also contributed to the growth of the biopesticide market in the country. Other factors have also played a pivotal role in this scenario. The growing demand from the consumer market for food with low toxic residue, as well as social and political pressures (e.g., regulatory agencies) for ecologically friendly and “low-risk” plant protection technologies that meet the requirements of sustainable agriculture, have driven this trend.

Over the past decades, Brazil has witnessed increasingly stringent regulatory measures for conventional chemical pesticides, including prohibitions or restrictions on existing products. Moreover, the scarcity of new chemical active ingredients with novel modes of action to combat resistance development in major pests and diseases, coupled with rising costs, stringent regulations, and the time involved in registering new chemical products, have also contributed to the increasing adoption of biopesticides. Consequently, there has been a growing surge in private investment and venture funding to support the development of new biological inputs. Technological advancements in formulations have also played a crucial role in introducing new biological products to the market.

## 6. Main Challenges and Opportunities for the Use of Biological Inputs in Brazil

The growth in the adoption of biological inputs in Brazil over the past years is evident, demonstrating an increasingly promising market. However, to ensure the success and widespread adoption of this technology, it is essential to overcome certain challenges. Firstly, investing in training and capacity-building programs for agricultural producers and consultants regarding the proper and effective use of biological inputs in farming is crucial. In this regard, providing access to specialized technical assistance in biological control could help producers choose, apply, and monitor biological inputs, thus ensuring their effectiveness.

Offering financial and tax incentives for products that adopt biological input consumption and usage practices in their crops would help reduce the initial adoption costs and make biological inputs more accessible to farmers. Moreover, partnerships between research institutions, universities, and specialized companies in agricultural biotechnology could promote benefits for the agricultural sector by fostering research and the continuous development of increasingly effective biological inputs. Furthermore, the collaborative efforts of these public and private institutions could assist in disseminating success cases of producers using biological inputs, serving as an effective way to encourage others to adopt these practices. This can be achieved through events, lectures, educational materials, and online platforms.

By implementing these measures in an integrated manner, it is possible to overcome the barriers currently limiting the adoption of biological inputs in Brazil, thus promoting their sustainable and confident use for the growth and modernization of the agricultural sector. In this context, biological inputs can reduce external dependence on imported inputs, lower production costs, and bring higher sustainability to agricultural production.

## 7. Concluding Remarks

The use of biobased products has shown extraordinary growth in recent years, especially in Brazil. These results come from a call for more sustainable production from all sectors of society, but require collaboration among producers, companies in the sector, and government agencies in Brazil.

Although there are still many doubts and uncertainties regarding producers in relation to organic products, especially with regard to their performance, effectiveness, or the correct way to use them; it is noted that there is an increase in the adoption of this technology in the field. Chemical products, traditionally used in agriculture, still continue to be adopted by large producers, but biological-based and plant-based inputs are reaching more and more spaces in crops, allowing efficient control of pests and diseases.

Companies in the agricultural sector are increasingly investing in the Brazilian market, seeking new and increasingly efficient products, as well as safe ones for application in agriculture. To foster the development and strengthen this market in the future, it is necessary to invest in Research and Development (R&D).

In this scenario, the actions of regulatory bodies are essential, and it is necessary to monitor the introduction of new products with regard to regulation and inspection in Brazil, as an efficient and safe technology for agricultural production in the country. Partnerships among biological input companies, research institutions, and government agencies could be established to promote and strengthen the biological input sector in Brazil.

## Figures and Tables

**Table 1:** Classification of pesticides according to Brazilian legislation [75].

Pesticide Product	Definition	Active Substances
*Biochemicals*	Products consisting of naturally occurring chemical substances with a nontoxic mode of action, used in the control of diseases or pests as agents that promote chemical or biological processes	(i) *Hormones and growth regulators*: Substances synthesized in one part of an organism that are transported to other sites where they exert behavioral control or regulate the growth of organisms. (ii) *Enzymes*: Naturally occurring proteins that catalyze chemical reactions, including peptides and amino acids, but not toxic proteins or those derived from genetically modified organisms. (iii) *Other products* may be included when they have an identical structure and functional identity to naturally occurring products and will be treated on a case-by-case basis according to current legislative norms.
*Semiochemicals*	Products consisting of chemical substances that evoke behavioral or physiological responses in recipient organisms and are used to detect, monitor, and control a population or biological activity of living organisms	(i) *Pheromones*: Substances that modulate communication between individuals of the same species (intraspecific responses). (ii) *Allelochemicals*: Biomolecules that influence the growth and development of biological and agricultural systems (interspecific responses).
*Biological Control Agents*	Living organisms, naturally occurring or obtained through genetic manipulation, introduced into the environment for the control of a population or biological activities of another organism considered harmful	(i) *Natural Enemies:* Organisms that naturally infect, parasitize, or prey on a specific pest, including parasitoids, predators, and entomopathogenic nematodes. (ii) *Sterile Insect Technique:* Involves releasing males that have been sterilized by ionizing radiation as a control method that can be used for pest suppression or eradication.
*Microbiological*		(i) *Microbiological Control Agents (MCAs):* Living micro-organisms, including viruses and those classified as genetically modified organisms, intended to prevent, destroy, repel, or mitigate any pest. (ii) *Biostimulants:* Micro-organisms and/or metabolites applied to stimulate physiological processes in plants that result in prevention or response to plant stress. They can promote the control of a population or the action of another harmful living organism. They can also act as defoliants, desiccants, growth stimulants, and growth inhibitors. (iii) *Metabolites:* A substance or class of substances produced by a population of cells, responsible either wholly or partially for the biological activity.

**Table 2:** Promising plants in the control of pests.

Plant	Family	Part Used	Class of Compounds	Target Pest	Reference
*Allium sativum*	Amaryllidaceae	Bulbs	Dimethyl trisulfide, diallyl disulfide, diallyl sulfide, diallyl tetrasulfide, 3-vinyl-[4 H]-1,2-dithiin, diallyl trisulfide, allyl trisulfide, 1,4 -dimethyl tetrasulfide, allyl disulfide, methyl allyl disulfide, and methyl allyl trisulfide	*Callosobruchus chinensis*	[94]
*Annona squamosa*	Annonaceae	Seeds	Caryophyllene oxide and acetogenins	*Chrysodeixis includens*	[95]
*Azadirachta indica*	Meliaceae	Seeds	Rotenone, deguelin, and tephrosin	*Helicoverpa armigera*	[96]
*Capsicum baccatum*	Solanaceae	Fruits	Capsaicinoids, carotenoids, and ascorbic acid	*Hovenia dulcis*	[97]
*Cymbopogon flexuosus*	Poaceae	Leaves	α-citral and β-citral	*Agrotis ipsilon*	[98]
*Eucalyptus camaldulensis*	Myrtaceae	Leaves	1,8-cineole, l-α-terpineol, and α-pinene	*Eragrostis plana*	[99]
*Lupinus albescens*	Fabaceae	Roots, stalks, leaves, and flowers	Stigmasterol, Ergosterol, Vitamin E, Methyl commate, Eicosanol, Epiergostanol, and Tetracosanol	*Fusarium oxysporum; Fusarium verticillioides*	[100]
*Nicotiana tabacum*	Solanaceae	Leaves	Alkaloids, Saponins, Diterphenes, Phytosterol, Flavonoids, and Phenols	*Callosobruchus maculatus*	[101]
*Ricinus communis*	Euphorbiaceae	Fruits	Carotenoid, Tocopherol, Tocotrienol, Phytosterol, and Phospholipid	*Melanaphis sacchari*	[102]
*Trichilia* spp.	Meliaceae	Fruits	Β-Sitosterol, Β-Amyrin, Stigmasterol, Campesterol, Sitostenone, Lupeol, Lupenone, Cryptomeridiol, and A-Amyrin	*Bemisia tabaci*	[103]
*Zingiber officinale*	Zingiberaceae	Rhizomes	Gingerol, Paradol, Shogaols, and Zingerone	*Bactrocera dorsalis*	[104]

## Data Availability

No new data were created or analyzed in this study. Data sharing is not applicable to this article.
